# Effectiveness of a multi-faceted intervention to deprescribe proton pump inhibitors in primary care: protocol for a population-based, pragmatic, cluster-randomized controlled trial

**DOI:** 10.1186/s12913-022-07496-3

**Published:** 2022-02-17

**Authors:** Jérôme Nguyen-Soenen, Cédric Rat, Aurélie Gaultier, Solène Schirr-Bonnans, Philippe Tessier, Jean-Pascal Fournier

**Affiliations:** 1 Département de Médecine Générale, Faculté de Médecine, Université de Nantes, Nantes, France; 2 SPHERE - UMR INSERM 1246, Université de Nantes, Université de Tours, Nantes, France; 3 Direction de la recherche, Plateforme de Méthodologie et Biostatistique, CHU Nantes, Nantes, France; 4 CHU de Nantes, Service Évaluation Économique et Développement des Produits de Santé, Nantes Université, Nantes, France

**Keywords:** Deprescriptions, Proton pump inhibitors, Patient outcome assessment, Primary care, Cluster analysis, Multi-faceted intervention

## Abstract

**Background:**

Inappropriately using proton pump inhibitors (PPI) is associated with severe adverse drug reactions and may have major consequences on healthcare costs. Deprescribing (the process by which a healthcare professional supervises the withdrawal of an inappropriate medication, to manage polypharmacy and improve outcomes) should be considered when an inappropriate PPI prescription is identified. Deprescribing interventions directed solely to prescribers have limited efficacy and are rarely targeted to patients. The aim of this trial is to assess the efficacy of a multi-faceted intervention with patients and general practitioners (GPs) to deprescribe PPI.

**Methods:**

We will conduct a pragmatic***,*** cluster-randomized, population-based, controlled trial in two regions of Western France. GPs with practices with over 100 patients, and their adult patient to whom over 300 defined daily doses (DDD) of PPIs have been dispensed in the year before baseline will be included. A total of 1300 GPs and 33,000 patients will be cluster-randomized by GPs practices. Three arms will be compared: i) a multi-faceted intervention associating a) a patient education brochure about PPI deprescribing sent directly to patients (the brochure was designed using a mixed-methods study), and b) a personalized letter with the Bruyere’s PPI deprescribing algorithm sent to their respective GPs, or ii) a single intervention where only the GPs received the letter and algorithm, or iii) no intervention.

The primary outcome will be PPI deprescribing, defined as the proportion of patients achieving at least a 50% decrease in the amount of PPI dispensed to them (DDD/year) at 12 months compared to baseline. Secondary outcomes will include incremental cost-utility ratio (using EQ-5D-5L scale and National Health Insurance’s database), acid rebound (using the Gastroesophageal Reflux Disease Impact Scale), and the patients’ attitudes towards deprescribing (using the French rPATD).

**Discussion:**

Based on previous trials, we anticipate more than 10% “successful PPI deprescribing” in the multi-faceted intervention compared to the single intervention on GPs and the control arm*.* The study has been funded through a national grant and will be launched in autumn 2020, for early results by the end of 2022.

**Trial registration:**

Clinicaltrials.gov NCT04255823; first registered on February 5, 2020.

**Supplementary Information:**

The online version contains supplementary material available at 10.1186/s12913-022-07496-3.

## Background

Proton pump inhibitors (PPI) are frequently prescribed inappropriately for excessive durations or inadequate indications in Europe [1, 2], the United-States [3] and France [4]. This inappropriate PPI use is associated with an increased risk of renal adverse events (acute interstitial nephritis, chronic renal failure) [5], *Clostridium difficile* diarrhea [6], pneumonia [7], fractures [8], dementia [9] or cardiovascular events [10]. Also, more recently, two studies discussed an associated, increased risk of colorectal cancer [11] and even mortality [12] with long-term PPI use compared to histamine-2 receptor antagonists. Additionally, excessive PPI use may have major consequences on healthcare costs. PPI reimbursements represented a total cost of $12 billion in the United-States, in 2015 [13] and £87 million in England, in 2018 [14], and is expected to rise since PPI prescriptions are continuously increasing [15].

Deprescribing is defined as “the process of withdrawal of an inappropriate medication, supervised by a healthcare professional with the goal of managing the polypharmacy and improving outcomes” [16]. Although some interventions have been developed to promote deprescribing of PPIs among physicians, pharmacist or nurses, they have limited efficacy [17]. However, multi-faceted interventions that include patients have demonstrated greater success in Australia, the United-States, and Europe [18–20].

### Trial objectives

The primary objective of this study is to assess if a multi-faceted intervention is superior to single intervention and usual care to increase PPI deprescribing. Three arms will be compared: i) a multi-faceted intervention with patients using a patient education brochure and with their general practitioners (GPs) using both a personalized letter and a deprescribing algorithm ii) a single intervention on GPs and iii) no intervention (usual care).

Secondary objectives are to assess if the multi-faceted intervention has an economic efficiency compared to control group by performing a cost-utility analysis, to determine the gastroesophageal reflux disease (GERD) symptom recurrence rate, the patients’ attitudes toward deprescribing, and the characteristics of patients engaging in the deprescribing process.

### Study hypothesis

We hypothesize that each of the deprescribing interventions will increase PPI deprescribing rates compared to usual care, and that the multi-faceted intervention (directed to both GPs and patients) will be superior.

## Methods

### Study design and setting

The trial is designed as a prospective, open-label, cluster-randomized parallel-controlled study within a 12-month period. The unit of randomization will be the GPs practice. This pragmatic population-based trial will be conducted in every eligible GP practice in two regions of Western France (Loire-Atlantique and Vendée).

### Study population

We will include both patients and their regular GPs if the GP has a regular patient population of more than 100 patients per year. Eligible patients must be affiliated to the French health national insurance system (Caisse Primaire d’Assurance Maladie, CPAM), and have been dispensed more than 300 defined daily doses (DDD) of PPI in the 12 months before baseline, estimated using the local health insurance reimbursement database (SIAM-ERASME). Those patients at risk of gastroduodenal lesions i.e. treated with nonsteroidal anti-inflammatory drugs (NSAIDs) and over 65 years old or treated with either corticosteroids or anticoagulants or platelet aggregation inhibitors will be excluded.

### Recruitment and allocation

Each GP practice and its associated patients will be defined as an individual cluster to avoid contamination bias between GPs and/or between patients [21]. These clusters will be randomized in three arms using a 1:1:1 ratio. Eligible patients will be identified using the local health insurance reimbursement database.

### Blinding

Blinding of patients and GPs will not be feasible given the pragmatic nature of the intervention. However, the research team will be blinded to the intervention designated to each arm.

### Interventions

**Fig. 1:**
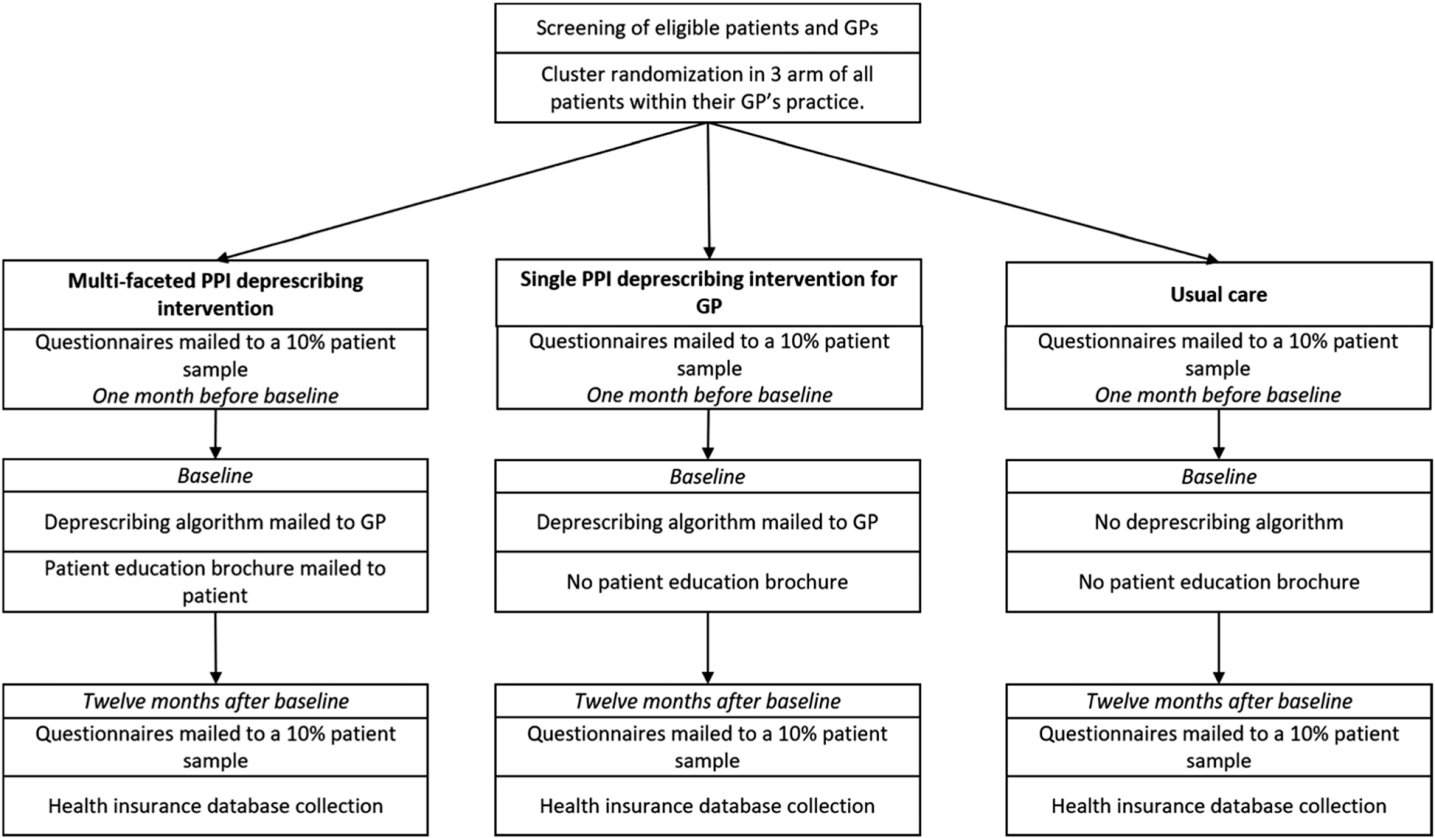
Study design

Two interventional arms and a control arm are planned (Fig. 1). The multi-faceted PPI deprescribing intervention will combine sending information to both patients and GPs. Patients will receive an education brochure and GPs will receive a personalized letter and deprescribing algorithm. The single PPI deprescribing intervention for GPs will consist of a personalized letter and deprescribing algorithm. The control arm will receive no deprescribing educational materials (usual care).

The selected deprescribing algorithm is an evidence-based clinical practice guideline [22] with the highest AGREE II score (unpublished data) [23], which was translated into French (supplementary material 1). We developed the patient education brochure using a previously reported mixed-method study [24] (supplementary material 2).

### Outcome measures

#### Primary outcome

PPI deprescribing will be defined as the proportion of patients achieving a 50% decrease in their PPI reimbursement (Defined Daily Dose (DDD)/year) at the end of the intervention (at 12 months compared to baseline).

#### Secondary outcomes

- Incremental cost-utility ratios: cost by quality-adjusted life-years (QALY) ratios comparing the intervention groups to control group. QALYs will be assessed from patient responses to the EuroQoL EQ-5D-5L questionnaire [25].
- GERD symptom recurrence using the GERD Impact Scale questionnaire [26].
- Patient attitude towards deprescribing using the French version of the Revised Patient Attitudes Toward Deprescribing (rPATD) [27], the *Attitude des patients envers la déprescription* (APaD) [28].
- Patient characteristics that succeed in deprescribing PPIs using data collected from health insurance reimbursement database.

### Data collection procedures

**Fig. 2:**
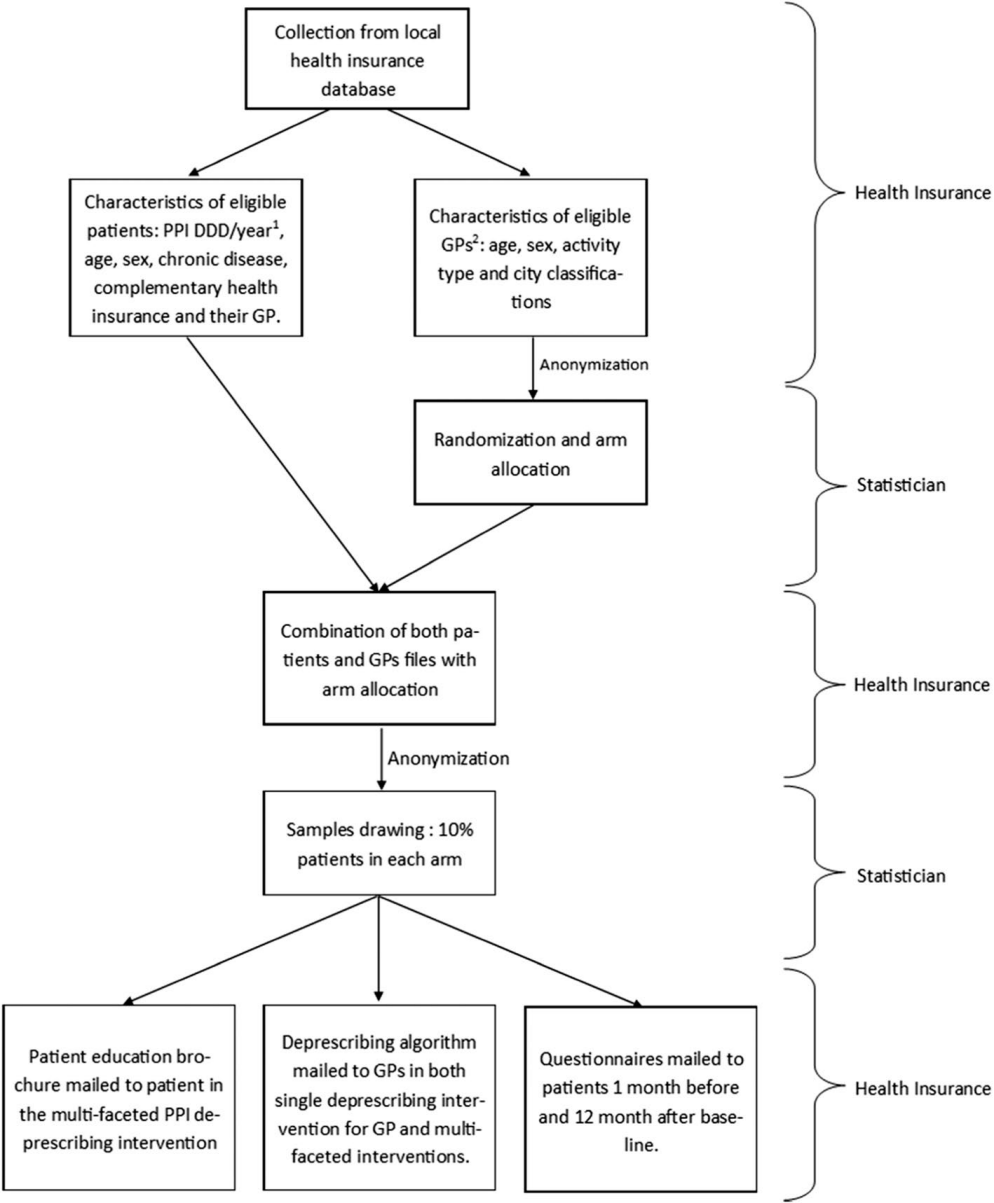
First collection from local health insurance database 1 month before baseline and data flow. ^1^PPI DDD/year: Proton pump inhibitors defined daily dose per year; ^2^GPs: General practitioners

Baseline patient variables (age, sex, PPI reimbursement, chronic disease, free complementary health insurance for economically vulnerable patients) and GP variables (age, sex, activity type and city classification) will be collected using the local health insurance reimbursement database (SIAM-ERASME) (Fig. 2).

Healthcare resource consumption over a one-year period will be collected by combining PPI use and medical expenses related to ambulatory care (histamine-2 receptor antagonists and antacids reimbursements, consultations, technical procedures and laboratory tests). These data will be collected using the local health insurance reimbursement database (SIAM-ERASME) 12 months after baseline.

Hospital service use will be collected using the French National Health Data System (*Système National des Données de Santé*, SNDS) [29].

Measuring health-related quality of life, GERD recurrence and attitudes toward deprescribing, requires sending self-administered questionnaires with pre-paid return envelopes and to scheduling phone calls to minimize missing answers. Thus, the analysis will only be performed on a 10% sample of the population.

Thus, the EQ-5D-5L, GERD Impact Scale and rPATD questionnaires will be mailed to a 10% patient sample in each arm, 1 month before and 12 months after baseline. Patients will return the three self-reported questionnaires to the National Health Insurance by post. Phone calls will be scheduled between 8 and 10 days later to remind patients to return the three questionnaires.

**Fig. 3:**
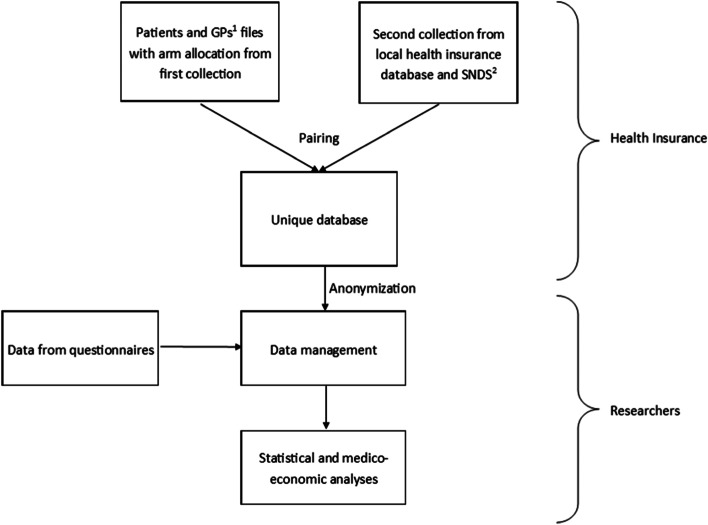
Second data collection from local health insurance database 12 months after baseline and data flow. ^1^GPs: General practitioners; ^2^SNDS: French national Health data System (*Système National des Données de Santé*)

The Health Insurance data managers will pair the data collected from these databases at the patient level, and proceed to data anonymization before transmission to the statistician (Fig. 3).

### Sample size and power

Preliminary analyses showed that around 33,000 patients, 1300 GPs and 684 practices would meet inclusion criteria in 2017, which corresponds to a mean of 1.9 GPs and 48.3 patients per practice. Using an intraclass correlation coefficient (ICC) of 0.05 thus lead to an inflation factor of 3.365. Literature data suggested a “natural” PPI deprescribing rate between 2 and 7% in the control arm [17, 20, 30].

Assuming an inflation factor of 3.365 and using a two- sided alpha of 0.05, we will have 90% power to detect a minimum true difference of 9.5% of PPI deprescribing rates between the multi-faceted intervention arm and the control arm. These estimates change slightly if different natural deprescribing rates and higher ICCs are assumed (Table 1).

**Table 1:** Sample size simulation

“Natural” PPI deprescribing rate	PPI deprescribing rate due to intervention	Patients per cluster	Intracluster Correlation Coefficient	Power
2%	17%	45	0.05	> 0.999
2%	17%	50	0.05	> 0.999
2%	17%	45	0.1	> 0.999
2%	17%	50	0.1	> 0.999
2%	10%	45	0.05	> 0.999
2%	10%	50	0.05	> 0.999
2%	10%	45	0.1	> 0.999
2%	10%	50	0.1	> 0.999
7%	17%	45	0.05	> 0.999
7%	17%	50	0.05	> 0.999
7%	17%	45	0.1	> 0.999
7%	17%	50	0.1	> 0.999
7%	10%	45	0.05	> 0.994
7%	10%	50	0.05	> 0.990
7%	10%	45	0.1	> 0.930
7%	10%	50	0.1	> 0.908

### Plan of statistical analyses

The analyses of primary and secondary outcomes will follow a modified intention-to-treat principle (mITT). All initially randomized patients and GPs and will be included in the analysis according to the group to which they were assigned, minus those who did not fulfil the inclusion criteria after randomization (for example patients with a PPI deprescribing occurring between randomization and intervention).

#### Primary analysis

PPI deprescribing rate at 12 months will be compared between each arm using a generalized linear mixed model. This model accounts for the random effect of GP practice.

#### Secondary analyses

##### Cost-utility analysis

The cost-utility analysis will aim to estimate incremental cost-utility ratios (ICUR) of cost per QALY comparing the multi-faceted intervention arm and single intervention on GP arm to control arm (usual care) as follows:

ICUR = [costs _deprescribing arm_ - costs _usual care arm_] / [QALYs _deprescribing arm_ - QALYs _usual care arm_].

The costs over a one-year time horizon will be estimated from a societal perspective excluding indirect costs such as production losses as recommended by French National Guidelines on the economic evaluation of health care programs [31]. To estimate costs, the healthcare resources consumed including, PPI consumption, medical consultations, examinations, laboratory analyses and hospitalization will be valued in monetary terms using conventional tariffs of the French National Health Insurance System.

Quality of life obtained will be measured with QALYS. QALYs are a numerical composite index combining information about quality of life and survival. They are constructed by weighting each year by a quality of life factor - called a utility score - typically ranging from 0 (death) to 1 (perfect health) such that a higher score represents a more preferred health state. They will be estimated from the patient answers to the EQ-5D-5L questionnaires sent at baseline and at 1 year. The EQ-5D-5L is a generic health status questionnaire that has five dimensions (mobility, self-care, usual activities, pain/discomfort, and anxiety/depression) each of which has five levels denoting increasing problems with the dimension under consideration. The patients answers will be converted to utility scores using the French published tariffs for the EQ-5D-5L [32]. Given the time horizon of 1 year, QALYs and costs will not be discounted.

Missing data will be handled using a multiple imputation method given that considering only complete cases would not correspond to an intention-to-treat analysis [33]. The ICUR, incremental costs and QALYs will be estimated using a seemingly unrelated regression method that accounts for the correlation between costs and QALYs. The analysis will also estimate the incremental net monetary benefit criterion (the monetized version of the ICUR) to construct acceptability curves that will present the probability for an intervention to be cost-effective for various social value of the willingness-to-pay for a QALY. Finally, sensitivity analyses will be conducted to assess the robustness of the results.

##### GERD recurrence

GIS questionnaire scores will be compared between each arm using a generalized linear mixed model to account for the random effect of GP’s practice.

##### Patient attitudes toward deprescribing

Cross-tabulations between the different rPATD questions and patient characteristics, and successful deprescribing at 12 months will be performed using Cochran-Armitage tests. Patient attitude measured from Likert scale will be compared between each arm with a generalized linear mixed model to account for the random effect of GP’s practice.

##### Characteristics of patients succeeding in PPI deprescribing

Association of patient characteristics (age, sex, socio-economic status, chronic disease, GP) and PPI deprescribing success will be tested with a generalized linear mixed model to account for the random effect of GP’s practice.

### Missing data

For the primary outcome analysis, data for patients lost to follow-up (i.e. death, moving outside the study region, or disaffiliation from the French health national insurance system) will be managed by a multiple imputation method (Generates Multivariate Imputations by Chained Equations) [34]. Patient characteristics used for this imputation will be the variables associated with the primary outcome or associated with the presence of missing data on the primary outcome. A sensitivity analysis will be performed by removing these patients from the analysis.

### Timeframe

Trial baseline will be planned in mid-November 2020 when deprescribing materials will be mailed to patients and GPs. The three patient reported outcome questionnaires will be mailed 1 month before and 12 months after baseline, mid-October 2020 and mid-November 2021 respectively. Data will be collected 12 months after baseline in mid-November 2021. Study schedule is detailed in the SPIRIT diagram (Table 2).

**Table 2:**
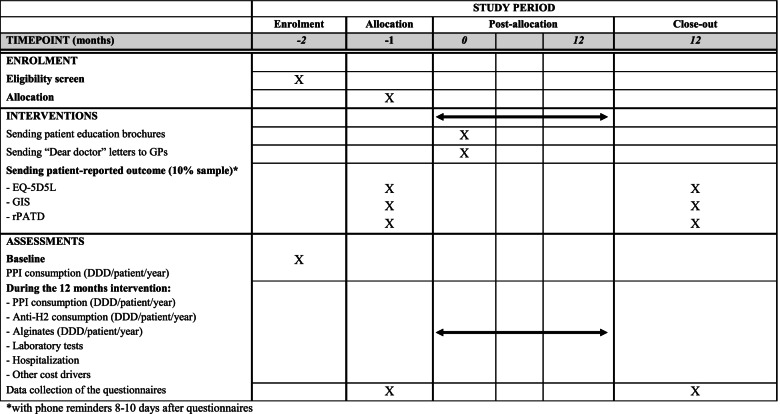
Study schedule (SPIRIT diagram of trial stages of enrolment, intervention, outcome assessment and evaluation)

### Ethics approval

This protocol has received ethics approval from the *Collège National des Généralistes Enseignants* ethics committee (#08062011).

## Discussion

### Study rationale

This trial will assess the effectiveness of three PPI describing strategies, with a view to applying them to deprescribing other medicines. In 2015, Clyne et al. reported that a multi-faceted intervention involving patient education materials for deprescribing reduced potentially inappropriate prescriptions in primary care by 25% more than control group, with a sustained effectiveness 1 year later [18]. In 2017, the multi-faceted Australian national quality improvement programs that also included patient education material contributed to reducing PPI use in older adults by 21% [19]. In 2020, several interventional cycles in the United-States included patient education pamphlets and discontinued inappropriate chronic PPI use by 30% within 12 months [20]. Based on these previous studies in Australia, Ireland and United-States, we anticipate that involving patients in the deprescribing process using an education brochure should improve the PPI deprescribing rate.

### Strengths and limitations

#### Strengths

Using the PRECIS-2 tool, 6 out of the 9 domains of the multi-faceted intervention fall into the pragmatic side of the pragmatic-explanatory continuum (supplementary material 3). The pragmatic approach involves intervention at the group level rather than the individual level and as such, cluster-randomizations are the most common in pragmatic trials [35]. Also, cluster-randomization are considered as the most appropriate to conduct deprescribing trials [36].

Our trial involves a large number of patients and GPs. It is, to our knowledge, the first population-based deprescribing trial in France. We expect to exhaust the eligible population ensuring a high statistical power for a minimal difference.

By using a 3-arm design, we will be able to assess which intervention is the most effective. The single intervention on GP versus usual care will detect the effectiveness of mailing the PPI deprescribing algorithm to GP. The multi-faceted intervention will measure an additional or a combined effect of the patient education brochure.

#### Limitations

Despite these strengths, our trial has several limitations. Firstly, regarding the deprescribing rate and the cost-utility analysis, our data will be collected from the health insurance reimbursement database. This database only estimates the consumption of medications through their reimbursements [37]. PPI consumption can be overestimated because real patient intake depends on adherence. Conversely, this database does not compile over-the-counter PPI but they are less used than reimbursable PPI [38].

Secondly, the secondary outcome on GERD recurrence is assessed by the GERD impact scale questionnaire at 12 months after baseline. Thus, we may miss GERD recurrence caused by an early acid rebound due to PPI withdrawal. However, sending this questionnaire during the intervention may influence patients’ perceptions, resulting in a participation bias on the primary outcome. We chose to keep our trial without further interventions during the study period. The GERD impact scale will only assess the GERD recurrence following PPI deprescribing.

Thirdly, neither patients nor their GP will be blinded because of the pragmatic trial design [39]. However, the participants will not be informed of the different interventions occurring during the year of the trial. Data managers and statisticians will be blinded to the allocation arm. Primary outcome (PPI deprescribing) is objective and not subject to interpretation.

Fourthly, because of logistical constraints, only a 10% sample of the population (nearly 3300 patients) will have the opportunity to answer questionnaires on secondary outcomes. The questionnaires will be sent by mail and the number of participants will depend on response-rate. However, the response-rate should be increased due to pre-paid return envelopes and amplified by phone reminders, as reported by Neve et al. for patient reported outcomes [40].

## Conclusion

Our trial is expected to demonstrate the effectiveness of the multi-faceted intervention involving both a patient education brochure and a personalized letter to GPs containing a PPI deprescribing algorithm. If so, this multi-faceted strategy for PPI deprescribing could be implemented more widely at the national level and for other potentially inappropriately prescribed medications.

### Trial status

First submission on ClinicalTrials.gov was February 5th 2020 and patient data are currently being collected from SNDS until the end of 2022.

### Adherence to reporting guidelines

The authors used the Standard Protocol Items: Recommendations for Interventional Trials (SPIRIT) checklist when they wrote this protocol [41] (Additional File).

## Supplementary Information

**Additional file 1: Supplementary material 1.** French PPI deprescribing algorithm (adapted from Bruyere research institute). **Supplementary material 2.** Patient education brochure for PPI deprescribing developed with a mixed-method study. **Supplementary material 3.** PRECIS-2 table. **Supplementary material 4.** Participant information note sent with questionnaires.

**Additional file 2.**

## Data Availability

Data sharing is not applicable to this article as no datasets were generated or analyzed during the current study yet.

## Abbreviations

DDD: Defined Daily Dose
GERD: Gastroesophageal Reflux Disease
GP: General Practitioner
ICC: Intracluster Correlation Coefficient
ICUR: Incremental Cost-Utility Ratios
mITT: Modified Intention-To-Treat
NSAIDs: Nonsteroidal Anti-Inflammatory Drugs
PPI: Proton Pump Inhibitors
QALY: Quality-Adjusted Life-Years
rPATD: Revised Patients’ Attitudes Toward Deprescribing

## References

[1] Muheim L, Signorell A, Markun S, Chmiel C, Neuner-Jehle S, Blozik E (2021). Potentially inappropriate proton-pump inhibitor prescription in the general population: a claims-based retrospective time trend analysis. Ther Adv Gastroenterol.

[2] Moriarty F, Bennett K, Cahir C, Fahey T (2016). Characterizing potentially inappropriate prescribing of proton pump inhibitors in older people in primary Care in Ireland from 1997 to 2012. J Am Geriatr Soc.

[3] Mafi JN, May FP, Kahn KL, Chong M, Corona E, Yang L (2019). Low-value proton pump inhibitor prescriptions among older adults at a large academic health system. J Am Geriatr Soc.

[4] Lassalle M, Le Tri T, Bardou M, Biour M, Kirchgesner J, Rouby F (2020). Use of proton pump inhibitors in adults in France: a nationwide drug utilization study. Eur J Clin Pharmacol.

[5] Lazarus B, Chen Y, Wilson FP, Sang Y, Chang AR, Coresh J (2016). Proton pump inhibitor use and the risk of chronic kidney disease. JAMA Intern Med.

[6] Furuya-Kanamori L, Stone JC, Clark J, McKenzie SJ, Yakob L, Paterson DL (2015). Comorbidities, exposure to medications, and the risk of community-acquired Clostridium difficile infection: a systematic review and Meta-analysis. Infect Contrl Hosp Epidemiol.

[7] Filion KB, Chateau D, Targownik LE, Gershon A, Durand M, Tamim H (2014). Proton pump inhibitors and the risk of hospitalisation for community-acquired pneumonia: replicated cohort studies with meta-analysis. Gut..

[8] Eom C-S, Park SM, Myung S-K, Yun JM, Ahn J-S (2011). Use of acid-suppressive drugs and risk of fracture: a meta-analysis of observational studies. Ann Fam Med.

[9] Gomm W, Fink A, Maier W, Doblhammer G, von Holt K, Thomé F (2016). Association of Proton Pump Inhibitors with Risk of dementia. JAMA Neurol.

[10] Shiraev TP, Bullen A (2018). Proton pump inhibitors and cardiovascular events: a systematic review. Heart Lung Circ.

[11] Abrahami D, McDonald EG, Schnitzer ME, Barkun AN, Suissa S, Azoulay L (2021). Proton pump inhibitors and risk of colorectal cancer. Gut.

[12] Xie Y, Bowe B, Yan Y, Xian H, Li T, Al-Aly Z (2019). Estimates of all cause mortality and cause specific mortality associated with proton pump inhibitors among US veterans: cohort study. BMJ.

[13] Peery AF, Crockett SD, Murphy CC, Lund JL, Dellon ES, Williams JL (2019). Burden and Cost of Gastrointestinal, Liver, and Pancreatic Diseases in the United States: Update 2018. Gastroenterology.

[14] Prescribing & Medicines Team, NHS Digital. Prescription Cost Analysis - England, 2018 (2019). Health and Social Care Information Centre.

[15] Othman F, Card TR, Crooks CJ (2016). Proton pump inhibitor prescribing patterns in the UK: a primary care database study. Pharmacoepidemiol Drug Saf.

[16] Reeve E, Gnjidic D, Long J, Hilmer S (2015). A systematic review of the emerging definition of ‘deprescribing’ with network analysis: implications for future research and clinical practice. Br J Clin Pharmacol.

[17] Wilsdon TD, Hendrix I, Thynne TRJ, Mangoni AA (2017). Effectiveness of interventions to Deprescribe inappropriate proton pump inhibitors in older adults. Drugs Aging.

[18] Clyne B, Smith SM, Hughes CM, Boland F, Cooper JA, Fahey T (2015). Sustained effectiveness of a multifaceted intervention to reduce potentially inappropriate prescribing in older patients in primary care (OPTI-SCRIPT study). Implement Sci.

[19] Pratt NL, Kalisch Ellett LM, Sluggett JK, Gadzhanova SV, Ramsay EN, Kerr M (2017). Use of proton pump inhibitors among older Australians: national quality improvement programmes have led to sustained practice change. Int J Qual Health Care.

[20] Nallapeta N, Reynolds JL, Bakhai S (2020). Deprescribing proton pump inhibitors in an academic, primary care clinic: quality improvement project. J Clin Gastroenterol.

[21] Torgerson DJ (2001). Contamination in trials: is cluster randomisation the answer?. BMJ.

[22] Farrell B, Pottie K, Thompson W, Al E (2017). Evidence-based clinical practice guideline for deprescribing proton pump inhibitors. Can Fam Physician.

[23] Brouwers MC, Kerkvliet K, Spithoff K, AGREE Next Steps Consortium ANS (2016). The AGREE Reporting Checklist: a tool to improve reporting of clinical practice guidelines. BMJ.

[24] Nguyen-Soenen J, Jourdain M, Fournier J-P. Development of patient education material for proton pump inhibitor Deprescribing: a mixed-methods study. Ann Pharmacother. 2021:10600280211046630. Online ahead of print. 10.1177/10600280211046630 34553640

[25] Self-complete on paper – EQ-5D. Available from: https://euroqol.org/eq-5d-instruments/eq-5d-5l-available-modes-of-administration/self-complete-on-paper/. [cited 2021 Oct 22]

[26] Louis E, Tack J, Vandenhoven G, Taeter C (2009). Evaluation of the GERD Impact Scale, an international, validated patient questionnaire, in daily practice. Results of the ALEGRIA study. Acta Gastroenterol Belg.

[27] Reeve E, Low L-F, Shakib S, Hilmer SN (2016). Development and validation of the revised patients’ attitudes towards Deprescribing (rPATD) questionnaire: versions for older adults and caregivers. Drugs Aging.

[28] Roux B, Sirois C, Niquille A, Spinewine A, Ouellet N, Pétein C (2021). Cross-cultural adaptation and psychometric validation of the revised patients’ attitudes towards Deprescribing (rPATD) questionnaire in French. Res Soc Adm Pharm.

[29] Tuppin P, Rudant J, Constantinou P, Gastaldi-Ménager C, Rachas A, de Roquefeuil L (2017). Value of a national administrative database to guide public decisions: From the système national d’information interrégimes de l’Assurance Maladie (SNIIRAM) to the système national des données de santé (SNDS) in France. Rev Epidemiol Sante Publique.

[30] Krol N, Wensing M, Haaijer-Ruskamp F, Muris JWM, Numans ME, Schattenberg G (2004). Patient-directed strategy to reduce prescribing for patients with dyspepsia in general practice: a randomized trial. Aliment Pharmacol Ther.

[31] Haute Autorité de Santé (2020). Methodology Guidance - Choices in Methods for Economic Evaluation.

[32] Andrade LF, Ludwig K, Goni JMR, Oppe M, de Pouvourville G (2020). A French value set for the EQ-5D-5L. PharmacoEconomics..

[33] Faria R, Gomes M, Epstein D, White IR (2014). A guide to handling missing data in cost-effectiveness analysis conducted within randomised controlled trials. Pharmacoeconomics..

[34] van Buuren S. Flexible imputation of missing data, Second Edition. Boca Raton: CRC Press; 2018. p. 444.

[35] Ford I, Norrie J (2016). Pragmatic trials. N Engl J Med.

[36] Clough AJ, Hilmer SN, Kouladjian-O’Donnell L, Naismith SL, Gnjidic D (2019). Health professionals’ and researchers’ opinions on conducting clinical deprescribing trials. Pharmacol Res Perspect.

[37] Palmaro A, Moulis G, Despas F, Dupouy J, Lapeyre-Mestre M (2016). Overview of drug data within French health insurance databases and implications for pharmacoepidemiological studies. Fundam Clin Pharmacol.

[38] Latry P, Molimard M, Bégaud B, Martin-Latry K (2010). How reimbursement databases can be used to support drug utilisation studies: example using the main French national health insurance system database. Eur J Clin Pharmacol.

[39] Godwin M, Ruhland L, Casson I, MacDonald S, Delva D, Birtwhistle R (2003). Pragmatic controlled clinical trials in primary care: the struggle between external and internal validity. BMC Med Res Methodol.

[40] Neve OM, van Benthem PPG, Stiggelbout AM, Hensen EF (2021). Response rate of patient reported outcomes: the delivery method matters. BMC Med Res Methodol.

[41] Chan A-W, Tetzlaff JM, Altman DG, Laupacis A, Gøtzsche PC, Krleža-Jerić K (2013). SPIRIT 2013 statement: defining standard protocol items for clinical trials. Ann Intern Med.

