# Supplementary material for: Effectiveness of a multi-faceted intervention to deprescribe proton pump inhibitors in primary care: protocol for a population-based, pragmatic, cluster-randomized controlled trial
Source: BMC Health Serv Res. 2022 Feb 17;22:219. doi: 10.1186/s12913-022-07496-3 (PMC8851828; doi:10.1186/s12913-022-07496-3)

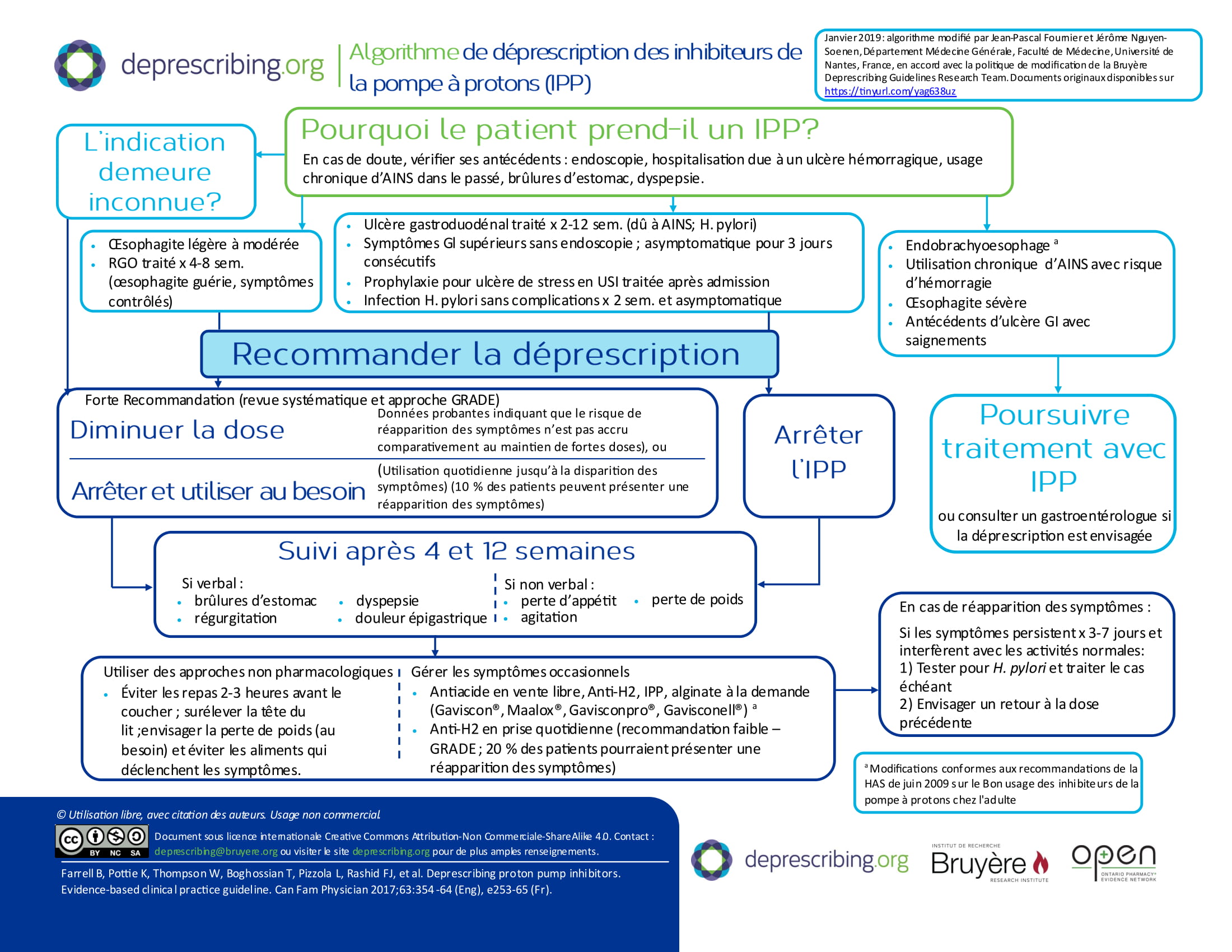
**Supplementary material 1 – French PPI deprescribing algorithm (adapted from Bruyere research institute)**

**Supplementary material 2 – Patient education brochure for PPI deprescribing developed with a mixed-method study**


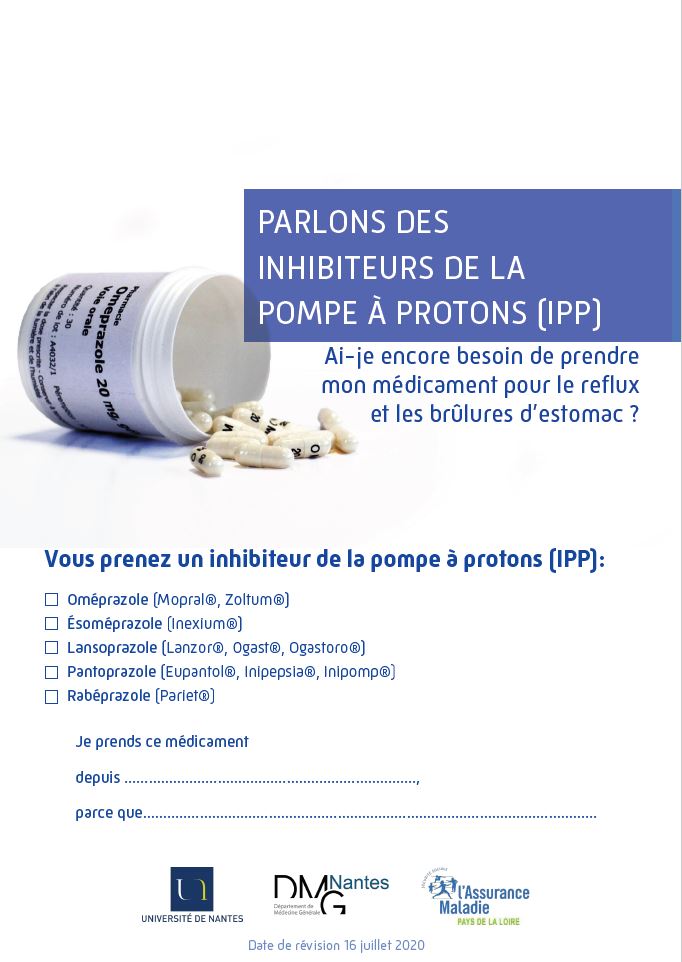

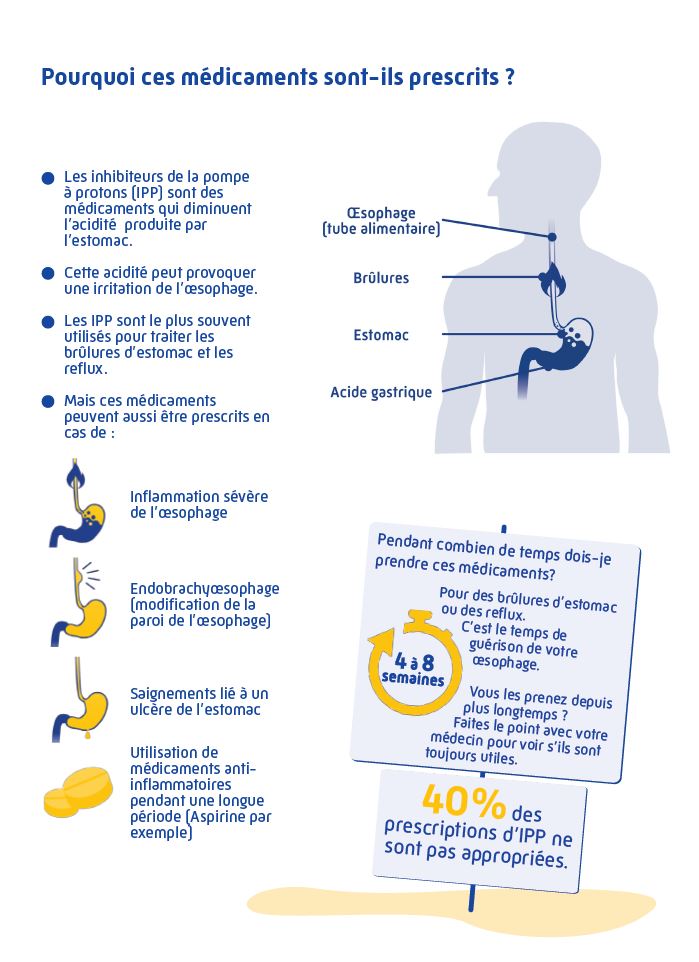

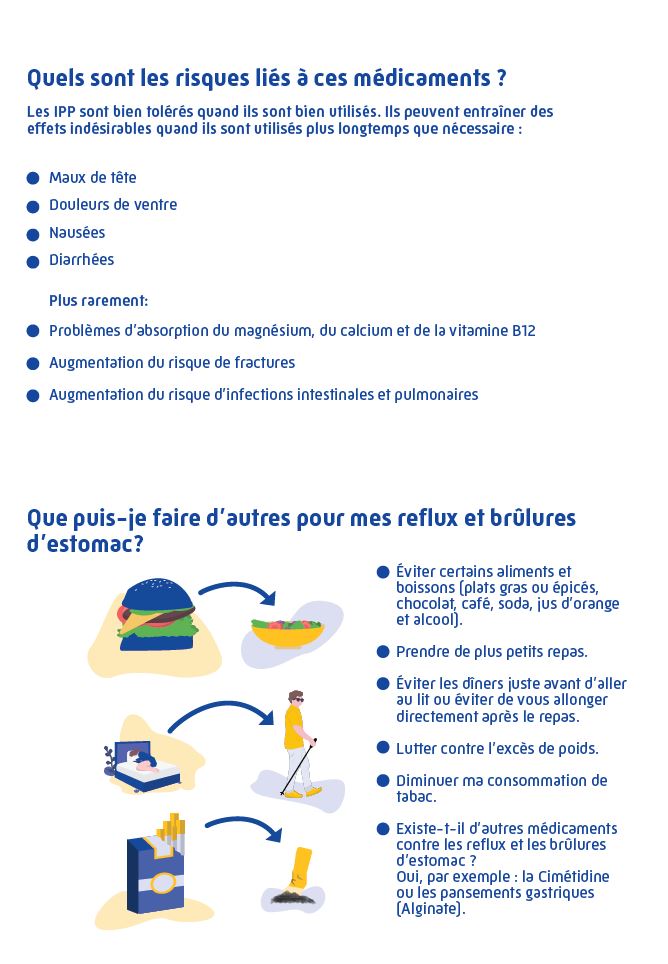

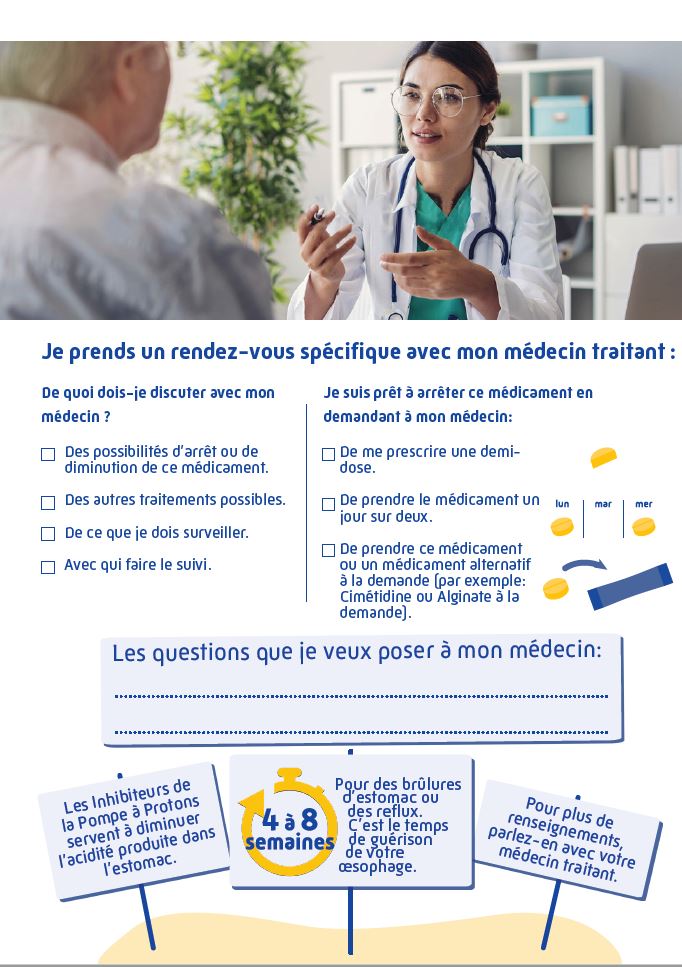


**Supplementary material 3 – PRECIS-2 table**

| **Domain** | **Score** | **Justification** |
| --- | --- | --- |
| **Eligibility** | 4 | Same as usual care but only adults |
| **Recruitment** | 1 | Targeted deprescribing materials |
| **Setting** | 5 | Same as usual care |
| **Organization** | 5 | Same as usual care |
| **Flexibility (delivery)** | 3 | Postal mail of deprescribing materials |
| **Flexibility (adherence)** | 5 | No exclusion based on adherence |
| **Follow-up** | 5 | No more than usual follow-up |
| **Primary outcome** | 3 | Theoretically accessible during consultations and relevant to participants but not assessed by clinical databases. |
| **Primary analysis** | 5 | Intention to treat |

**Supplementary material 4 - Participant information note sent with questionnaires**


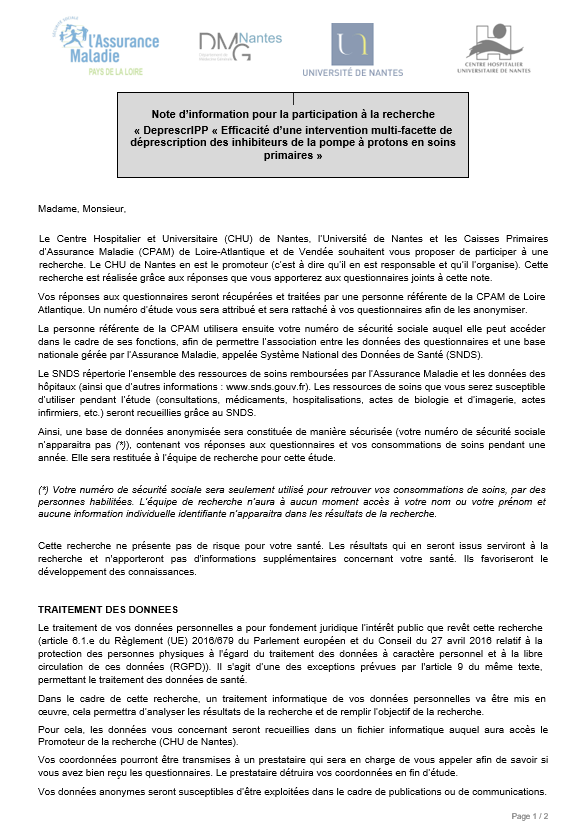


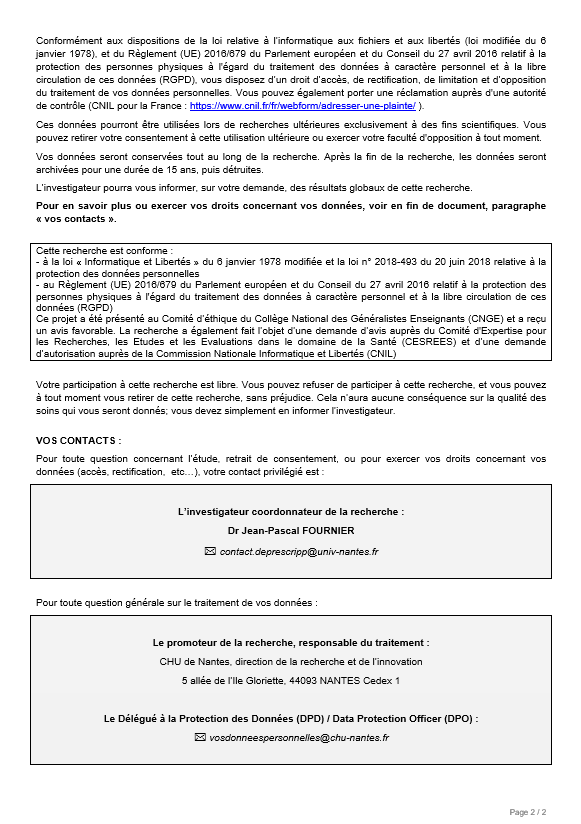

Supplement: Supplementary file 1 — Additional file 1: Supplementary material 1. French PPI deprescribing algorithm (adapted from Bruyere research institute). Supplementary material 2. Patient education brochure for PPI deprescribing developed with a mixed-method study. Supplementary material 3. PRECIS-2 table. Supplementary material 4. Participant information note sent with questionnaires. [file 12913_2022_7496_MOESM1_ESM.docx]
